# Human Papillomavirus and Oropharyngeal Squamous Cell Carcinoma: A Case-Control Study regarding Tobacco and Alcohol Consumption

**DOI:** 10.4061/2011/806345

**Published:** 2011-07-12

**Authors:** F. Farshadpour, S. Konings, E. J. M. Speel, G. J. Hordijk, R. Koole, M. van Blokland, P. J. Slootweg, J. A. Kummer

**Affiliations:** ^1^Department of Otorhinolaryngology and Head and Neck Surgery, University Medical Center Utrecht, P.O. Box 85500, 3508 GA Utrecht, The Netherlands; ^2^Department of Molecular Cell Biology and Pathology, Grow-School for Oncology & Developmental Biology, Maastricht University Medical Center, P.O. Box 5800, 6202 AZ Maastricht, The Netherlands; ^3^Department of Oral and Maxillofacial Surgery, University Medical Center Utrecht, P.O. Box 85500, 3508 GA Utrecht, The Netherlands; ^4^Department of Pathology, Radboud University Nijmegen Medical Centre, P.O. Box 9101, 6500 HB Nijmegen, The Netherlands; ^5^Department of Pathology, University Medical Center Utrecht, P.O. Box 85500, 3508 GA Utrecht, The Netherlands

## Abstract

We aimed to determine the role of HPV in the pathogenesis and outcome of oropharyngeal squamous cell carcinoma (OSCC) in lifelong nonsmoking and nondrinking patients. A case-case analysis was performed to compare the presence of HPV-DNA in tumor cells of 16 nonsmoking and nondrinking with 16 matched smoking and drinking patients (matching criteria: age at incidence, gender, tumor sublocation, tumor stage). HPV was detected using 2 PCR tests, FISH analysis, and p16^INK4A^ immunostaining. Nonsmoking and nondrinking patients had more HPV-positive tumors than smoking and drinking patients (*n* = 12; 75% versus *n* = 2; 13%; *P* < 0.001). All HPV-positive tumors showed p16^INK4A^ overexpression, and 1 HPV-negative tumor had p16^INK4A^ overexpression, (*P* < 0.001). Overall survival and disease-specific survival were higher for HPV-positive compared to HPV-negative cases (*P* = 0.027, *P* = 0.039, resp.). In conclusion, HPV is strongly associated with OSCC of nonsmoking and nondrinking patients. Specific diagnostic and therapeutic actions should be considered for these patients to achieve a better prognosis.

## 1. Introduction

The most important risk factors for developing head and neck squamous cell carcinoma in the Western countries are consumption of tobacco and alcohol [1]. However, there is a small population of nonsmoking and nondrinking patients with head and neck squamous cell carcinoma, so other risk factors may be important [2]. Substantial evidence has shown that oncogenic human papillomavirus (HPV), which is the primary cause of uterine cervical cancer, is etiologically involved in the development of head and neck squamous cell carcinoma [3–10]. 

It is estimated that up to 15–20% of all head and neck squamous cell carcinomas are associated with high-risk HPV infection [3–10]. This prevalence varies broadly, depending on the sublocation of the tumor, the studied population, the detection method, and the type of specimen used [4–10]. The highest rates of HPV-DNA (up to 70%) have been found in oropharynx squamous cell carcinomas (OSCCs), especially the tonsils. HPV type 16 has been detected in 90–95% of HPV-related OSCC, HPV-18 in some cases, and HPV type 31, -33, and -35 in considerably less cases [7, 9–13].

In the pathogenesis of HPV-related cancer, integration of the viral genome into the cellular DNA and, as a result, upregulation of the viral oncoproteins E6 and E7 seem to be crucial events. These oncoproteins subsequently cause dysfunction of amongst others tumor suppressor proteins, p53 and pRb, respectively, leading to cell proliferation, impaired apoptosis, and ultimately chromosome instability [14]. 

Immunohistochemical detection of p16^INK4A^ overexpression, a product of tumor suppressor gene CDKN2A, has been associated with HPV-related head and neck squamous cell carcinoma and in some studies used as a surrogate biomarker for HPV detection [5, 15, 16]. Recent studies have characterized a subset of HPV-related OSCC in which p16^INK4A^ overexpression predicts the presence of oncogenic HPV infection and identifies those with a better prognosis [17, 18]. Moreover, deletion of the CDKN2A locus together with functional inactivation of the tumor suppressor protein p16^INK4A^ have been detected in head and neck squamous cell carcinoma without a relationship with HPV infection [19, 20].

 HPV-positive head and neck squamous cell carcinomas are predominantly poorly differentiated and show a characteristic basaloid morphology in comparison with HPV-negative tumors [4, 6]. Furthermore, patients with HPV-positive tumors are less likely to consume large amounts of tobacco and alcohol [9, 15, 21, 22] and seem to have a better response to radiotherapy and a favorable survival rate [4, 11, 18, 23, 24]. So there are signs that these tumors form a separate entity within the heterogeneous group of head and neck squamous cell carcinomas.

The correct determination of HPV's involvement in the pathogenesis and prognosis of OSCC is dependent on several patient- and tumor-related cofactors, such as tobacco and alcohol use, TNM stage, and treatment modality. Although most investigators have found a trend between HPV and lesser amount of tobacco and alcohol use, the definitions of the used amounts are not always clear. Furthermore, to date, no matched analysis with smoking and drinking patients has been performed. In addition, previous studies have often used only one assay to determine the biological association of HPV infection with tumorigenesis. 

In this study, we aimed to determine the role of HPV in carcinogenesis and disease outcome for nonsmoking and nondrinking patients with OSCC. Therefore, we performed a case-case study of a well-defined population of 16 nonsmoking and nondrinking and 16 matched, smoking and drinking patients with OSCC for the presence of HPV DNA and overexpression of biomarker p16^INK4A^. The presence of HPV DNA was analysed using three different methods, that is, fluorescence in situ hybridization (FISH) and two polymerase-chain-reaction- (PCR-) based assays (Amplicor and Linear Array HPV detection kits). 

## 2. Material and Methods

Patients were selected from a database at the University Medical Center Utrecht, in which all patients with newly diagnosed head and neck squamous cell carcinoma are prospectively registered. Between 1980 and 2004, 4607 patients were entered in the database. This database contains information on patient characteristics, risk factors, tumor classification, treatment modalities, and follow-up data including number of recurrences and subsequent primary tumors. Patients were classified as nonsmoking and nondrinking (*n* = 198), when they had no history of smoking tobacco and alcohol consumption. Patients were classified as smoking and drinking (*n* = 2181), when they actively smoked tobacco and consumed alcohol. Former smokers or drinkers were not included. All patients were treated according to institutional protocols, and final decision was made in consultation with the patient. Follow-up time (in months) was considered from the date of diagnosis (i.e., first proven biopsy) to the date of death or date of last followup (January 1, 2009). Seventeen nonsmoking and nondrinking patients with a primary head and neck squamous cell carcinoma located in the oropharynx (ICD codes 141.0, 145.3, 145.4, 146.0, 146.1, 146.2, 146.3, and 146.6) were found in the database of which 16 were selected because of absence of tumor tissue in 1 case. These patients were matched with smoking and drinking patients on gender, age (±5 years), sublocation of tumor, and tumor stage. A case-case analysis was performed to compare the prevalence of HPV DNA and overexpression of p16^INK4A^ in both groups. 

### 2.1. Tissue Specimens

32 formalin-fixed, paraffin-embedded (FFPE) tumor tissue blocks from either biopsy or surgical resection specimens were obtained. Two experienced head and neck pathologists (JAK, PS) examined H&E-stained slides to select the areas in which tumor cells were present and evaluated the morphological appearances. Both pathologists were blinded to the smoking and drinking status. Tumor grade was recorded as well, moderate, or poor according to the criteria of the World Health Organization [25]. In addition, tumors were assessed for the absence or presence of hyperkeratosis, vasoinvasive and perineural growth, and typical basaloid features, that is, small, dark cells with scant cytoplasm, hyperchromatic nuclei, marked mitotic activity, a predominant lobular pattern of growth, and the absence of prominent keratinisation [26].

### 2.2. HPV Analysis

#### 2.2.1. DNA Isolation and PCR Analysis

For DNA extraction tumor, areas from FFPE slides were isolated by microdissection. After deparaffinization, the tissue fragments were digested in 150 *μ*L 50 mM Tris/HCL (pH 8.0) 0,5% (v/v) Tween-20 with proteinase K (final concentration 2 mg/mL). After 1 hour incubation at 56°C, the lysates were boiled to inactivate the proteinase K and subsequently centrifuged. Supernatants were transferred into clean eppendorf tubes and directly used for PCR. PCR was performed using the Amplicor HPV Test kit (Amplicor HPV Amplification kit: 03610799 190, Amplicor HPV Detection kit: 03610799 190, Amplicor HPV Controls Kit: 03610756 190; Roche, Basel, Sz) as well as the Linear Array HPV Genotyping Test (Linear Array HPV Genotyping Kit: 03378179 190, Linear Array HPV Detection Kit: 208693; Roche). Both tests were carried out according to the manufacturer's recommended protocol including positive and negative controls. The Amplicor test is a qualitative in vitro test which uses amplification of target DNA by PCR and nucleic acid hybridization for the detection of high-risk HPV DNA genotypes (i.e., HPV types 16, 18, 31, 33, 35, 39, 45, 52, 56, 58, 59, 66, and 68). It uses primers to define a sequence of nucleotides within the L1 region of the HPV genome that is 150 base-pair (bp) long. This test also features a concurrent isolation and amplification of the human *β*-globin gene to assess DNA integrity for each tested specimen. The Linear Array test uses the same detection technique; however, it targets an HPV genome sequence of 450 bp and is able to detect high-risk (same types as mentioned above) as well as low-risk HPV-DNA (i.e., HPV types 6, 11, 40, 42, 43, and 44). 

#### 2.2.2. FISH

FISH was performed on 4 *μ*m thick tissue sections as described previously [5, 15]. Briefly, sections were deparaffinized, pretreated with 85% formic acid/0.3% H_2_O_2_, 1 M NaSCN, and 4 mg/mL pepsin in 0.02 M Hcl, postfixed in 1% formaldehyde in PBS, dehydrated in an ethanol series, and hybridized with a digoxigenin-labeled HPV 16-specific probe (PanPath, Amsterdam, The Netherlands) according to the manufacturer's instructions. After hybridization, the preparations were washed stringently in 50% formamide, 2×SSC, pH 7.0 at 42°C (2 times 5 min). The probes were detected by application of mouse antidigoxin (Sigma, St. Louis, MO), peroxidase-conjugated rabbit antimouse IgG, and peroxidase-conjugated swine antirabbit IgG (both Dako; Glostrup, Dk) and visualized by a peroxidase reaction using rhodamine-labeled tyramide. Preparations were mounted in Vectashield (Vector Laboratories, Burlingame, Calif, USA) containing 4,6-diamidino-2-phenyl indole (DAPI; Sigma: 0.2 ug/mL). Microscope images were recorded with the Metasystems Image Pro System (black and white CCD camera; Sandhausen, Germany) mounted on top of a Leica DM-RE fluorescence microscope equipped with DAPI and rhodamine filters. Evaluation of nuclear hybridization signals was performed by two investigators (FF and EJMS) according to the previously described criteria [15]: punctate and/or diffuse signals throughout the nucleus indicating integrated and episomal HPV DNA, respectively, and granular FISH pattern if >1 nuclear signals, varying significantly in size and intensity, were observed. Control hybridizations were performed as described previously [15].

#### 2.2.3. Immunohistochemical Detection of P16^INK4A^

4 *μ*m thick tissue sections were deparaffinized with xylene and rehydrated by serial ethanol dilutions. Endogenous peroxidase activity was blocked by incubation for 30 minutes with 0.3% (v/v) H_2_O_2_ in methanol followed by antigen retrieval by boiling in 0.01 M sodium citrate buffer pH 6 for 15 minutes in a microwave oven. Slides were then incubated with a p16^INK4A^-specific primary mouse monoclonal antibody (Neomarkers, Fremont, USA) and diluted 1 : 160 for one hour at room temperature followed by a secondary visualisation reagent for 45 minutes (Powervision Goat-anti-Mouse/Rabbit/Rat labelled with horseradish peroxidase, ImmunoLogic, ImmunoVision Technologies, Brisbane, USA). After each incubation step, slides were washed in phosphate-buffered saline containing 3% (w/v) BSA. Peroxidase activity was visualized by incubation with diaminobenzidine/H_2_O_2_, and cell nuclei were counterstained with hematoxylin. All p16^INK4A^-positive cases were assessed for nuclear and/or cytoplasmic staining pattern. The staining patterns were scored semiquantitatively for the percentage of p16^INK4A^-positive tumor cells. The sections were graded as positive (+) when at least 75% of the tumor cells showed p16^INK4A^ positivity and as negative (−) when no staining was visible. Only one case (Table 2) showed 25% p16^INK4A^-positive tumor cells and was considered as ±. 

#### 2.2.4. Statistics

The association between HPV status and other variables was tested using Chi-square and Fisher's exact test. Disease-specific survival (i.e., death due to primary tumor, tumor recurrence, or subsequent primary tumor) and overall survival (i.e., mortality due to all causes) were determined for HPV-positive and HPV-negative cases, nonsmoking and nondrinking and smoking and drinking groups, and for cases with and without p16^INK4A^ overexpression using a univariate approach (i.e., Kaplan-Meier) method as patients were matched on possible confounding factors. Estimated survival curves were compared using log-rank test. A *P* value ≤0.05 was considered statistically significant.

## 3. Results

Sixteen nonsmoking and nondrinking patients with OSCC were matched with 16 smoking and drinking patients according to the above-mentioned criteria. The smoking and drinking patients used the following amounts of tobacco and alcohol at the time of diagnosis: 2–4 units of alcohol/day (*n* = 10), 5–9 units of alcohol/day (*n* = 4), and >9 units of alcohol/day (*n* = 2); ≤20 cigarettes/day (*n* = 5) and >20 cigarettes/day (*n* = 11). For 2 nonsmoking and nondrinking patients, the best possible match was disease stage IVA instead of III. The incidence dates ranged from 1980 to 2005.  Table 1 summarizes the basic clinical characteristics of all cases. 

HPV status for all cases was determined using two PCR-based test kits and FISH analysis (Table 2). The Amplicor PCR test showed 12 HPV-positive and 15 HPV-negative cases and was in 5 cases inconclusive due to negative *β*-globin gene results. The Linear Array PCR test showed 7 HPV-positive and 19 HPV-negative cases, and 6 cases that were inconclusive due to negative *β*-globin gene results. The FISH analysis revealed 12 HPV 16-positive cases of which 1 with a very low signal intensity (4B), and 19 cases without a detectable signal and 1 case which was inconclusive due to insufficient tissue material. Eight of the FISH-positive cases showed punctate signals in the tumor cell nuclei indicating integrated HPV DNA, and 4 showed granular nuclear staining. Based on these outcomes (see also discussion), we determined the HPV status as follows: 12 of 16 nonsmoking and nondrinking cases (75%) had a positive HPV status versus 2 of 16 smoking and drinking controls (12.5%, *P* < 0.001, Tables 2 and 3). 

Immunohistochemical analysis for biomarker p16^INK4A^ was detected as shown in Tables 2 and 3. p16^INK4A^overexpression (at least 75% of cells with positive staining) was found in 14 cases (44%), in 1 case (1B) 25% of cells stained positive (3%) and 17 cases (53%) were negative. All positive cases had strong nuclear as well as cytoplasmic staining except case 16B which showed predominantly cytoplasmatic staining. All HPV-positive cases had p16^INK4A^overexpression, whereas 17 of 18 HPV-negative cases had no detectable p16^INK4A^ (*P* < 0.001, Table 3). 

The associations between HPV status and tumor subsite, T- or N-classification, tumor stage, year of initial diagnosis, treatment, and vasoinvasive and perineural growth were not significant (Table 3). In contrast, HPV-positive tumors showed significantly less often keratinisation (*P* = 0.025) and more often basaloid features (*P* = 0.039, Table 3). Tumor recurrence was found in 3 HPV-positive (2 locoregional and 1 distant) and 3 HPV-negative cases (all locoregional) and in 2 nonsmoking and nondrinking and 4 smoking and drinking patients. Second primary tumor was found in 1 HPV-positive (in the oral cavity) and 4 HPV-negative cases (1 oral cavity, 3 oropharynx, and 1 lung) and in 1 nonsmoking and nondrinking patient and 5 smoking and drinking patients (Table 3, no significant correlations).

### 3.1. Survival Data

**Figure 1:**
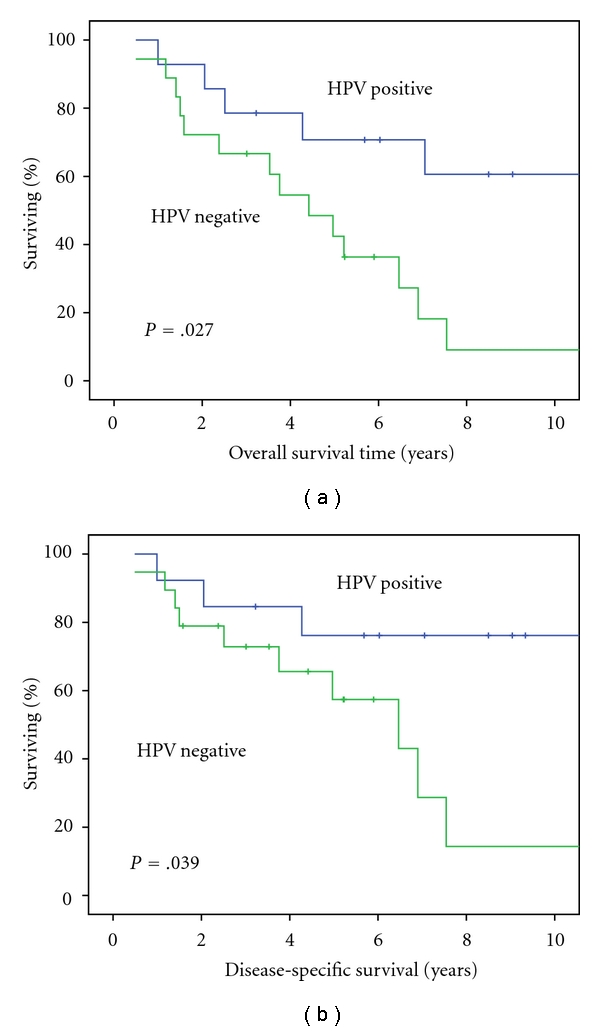
(a) Overall survival for HPV-positive compared to HPV-negative cases. (b) Disease-specific survival for HPV-positive compared to HPV-negative cases.

Follow-up time ranged from 5.9 to 182.1 months. Median follow-up time was 61.1 months. The 5-year overall and disease-specific survival for all cases was 53% and 64%, respectively. Cause of death in 20 deceased patients was as follows: due to primary tumor (*n* = 4; 1 nonsmoking and nondrinking, 3 smoking and drinking), other causes (*n* = 7; 4 nonsmoking and nondrinking, 3 smoking and drinking) of which 5 cardial and 2 pulmonary disease, recurrent disease (*n* = 6; 2 nonsmoking and nondrinking, 4 smoking and drinking), and second primary tumor (*n* = 3 smoking and drinking). For HPV-positive and HPV-negative cases, the 5-year overall survival was 71% and 42%, and 5-year disease-specific survival was 76% and 57%, respectively. Overall and disease-specific survival were both significantly higher for HPV-positive compared to HPV-negative cases (*P* = 0.027, *P* = 0.039, resp., Figure 1), for nonsmoking and nondrinking patients compared to the smoking and drinking counterparts (*P* = 0.037, *P* = 0.013, resp.) and for cases with p16^INK4A^ overexpression compared to those without detectable p16^INK4A^ overexpression (*P* = 0.028, *P* = 0.030, resp.). 

## 4. Discussion

To date, this study is the first that analyses the role of HPV in the pathogenesis and clinical behavior of OSCC in nonsmoking and nondrinking patients in comparison with matched smoking and drinking patients. HPV was strongly associated with OSCC in the absence of tobacco and alcohol use. HPV was found in 86% of the nonsmoking and nondrinking patients compared to 22% of the smoking and drinking patients. Our results are consistent with other studies although they mostly have shown this association separately in a group of nonsmokers or in a group of nondrinkers. Lindel et al. found HPV in 62 percent of nonsmokers and 38 percent of nondrinkers with oropharyngeal tumors [9]. Tachezy et al. demonstrated HPV-positive oropharynx and oral cavity tumors in all nonsmokers and 69% of nondrinkers, and in a recent study, nonsmoking and nondrinking patients with OSCC were reported to be 6.1 times more likely to be infected with high-risk HPV [22, 27]. One could hypothesize that smoking and drinking are independent risk factors and that the effect of HPV is enriched in the absence of these risk factors [28]. Increasing evidence shows a particular risk factor profile for HPV-related head and neck squamous cell carcinoma with not only less consumption of tobacco and alcohol but also a different sexual behavior and higher use of marijuana in mostly younger patients (<55 years [28]) compared to non-HPV-associated head and neck squamous cell carcinoma [21, 29, 30]. We do not have patient data regarding sexual behavior and use of drugs in our studied population. 

Additional characteristics of our studied HPV-positive tumors included the presence of basaloid features and lack of keratinisation which has been reported by previous studies [4, 6]. Likewise in this study as well as numerous other studies, HPV-related tumors proved to be associated with not only a better overall survival but also a better disease-specific survival [4, 9, 16, 23, 24]. The underlying mechanism for this prognostic effect of HPV is unclear. Although only one HPV-positive case had a second primary tumor compared to three HPV-negative cases, this difference was not significant. Also no correlation was found between recurrent disease or different treatment modalities and HPV positivity. Nevertheless, a better response on treatment like an increased sensitivity for radiotherapy possibly due to remaining amounts of p53 function in HPV-associated tumors might also explain the favorable prognosis. Worden et al. found induction chemotherapy followed by chemoradiotherapy to be an effective treatment in especially HPV-positive OSCC [31]. So it seems important to recognize patients with HPV-related head and neck squamous cell carcinoma to customize therapeutic decisions. Moreover, combination of HPV with recently identified prognostic indicators such as loss of chromosome 16q and the presence of p21^CIP1/WAF1^or nuclear survivin expression holds further promise to select patients for this purpose [19].

We also found better overall and disease-specific survival for nonsmoking and nondrinking cases and those with p16^INK4A^ overexpression compared to their counterparts. We consider these results to be related to HPV positivity. In another recent study by our studygroup regarding disease outcome for all head and neck squamous cell carcinoma in our center, we found no difference in survival between those who smoke and drank and those who did not [32]. 

Some controversy exists concerning the most reliable way to determine biologically relevant HPV infection in FFPE tissue. Therefore, it has been proposed to use at least more than one method to identify a firm association of the virus with the tumor cells. Most studies agree upon the use of the surrogate marker p16^INK4A^ followed by a HPV-specific test, such as HPV DNA PCR [16, 18], HPV E6 RT-PCR [17], or HPV FISH [15, 33]. We used four methods to detect the HPV status, that is, p16^INK4A^ immunostaining, PCR using two different test kits, and FISH analysis, which strongly correlated with each other. In 4 cases (9A, 12A, 12B, and 16A), the *β*-globin gene could not be amplified by both PCR tests; hence, the FISH data were used to determine the HPV status, which corresponded with the presence of p16^INK4A^ overexpression in case of HPV positivity. Nevertheless, also some discrepancies were found between the different tests used. In cases 5A and 2A, the Amplicor test was positive for HPV, whereas the Linear Array test was negative, probably due to the large fragments that need to be amplified in the latter assay. As a consequence, the Amplicor and the FISH results were used to proof HPV positivity for these cases. In case 1A, PCR revealed the presence of HPV DNA of types 33/52, 33, 35, and 58 with corresponding p16^INK4A^ overexpression, which explains the negative outcome of the HPV type 16-specific FISH analysis. Only in cases 1B and 4B, FISH analysis did not correlate with PCR and p16^INK4A^ immunostaining, and in these cases, we decided to consider a positive p16^INK4A^ and PCR status as signs for HPV positivity. However, the opposite may also be true as one considers the very high sensitivity of HPV DNA PCR, which may lead to false-positive results [34], as well as the fact that p16^INK4A^ can be overexpressed without the presence of HPV, for example, case 16B and a study by Hafkamp et al. [15]. On the other hand, the p16^INK4A^ staining pattern in case 16B was purely cytoplasmic in contrast to the other p16^INK4A^-positive cases in which cytoplasmic and nuclear pattern was seen. This may point to other reasons than HPV for upregulation of this biomarker. Furthermore, the results as mentioned in Table 3 and survival curves would not be affected by opposite results of cases 1B and 4B. 

We conclude that HPV is strongly associated with oropharyngeal tumors, especially in lifelong nonsmokers and nondrinkers. With better and more valid detection techniques, it is likely that these patients will be recognized as a specific entity within the heterogeneous group of head and neck cancer. Diagnostic and therapeutic actions will then be more focussed on this distinct group and may lead to better prognosis. 

## Figures and Tables

**Table 1:** Basic characteristics of all cases.

	Nonsmoking and nondrinking	Smoking and drinking
	*n*	**n**
Gender		
Male	3	3
Female	13	13
Age at tumor incidence (years)		
Mean	64.8	63.0
Range	45–83	50–78
Tumor stage		
II	3	3
III	6	4*
IVA	7	9*
Year of initial diagnosis		
1982–1986	2	1
1987–1991	2	3
1992–1996	6	5
1997–2001	2	5
2002–2006	4	2
Tumor location (ICD-code)		
Base of tongue (141.0)	6	6
Tonsil (146.0)	5	5
Tonsillar fossa (146.1)	3	3
Vallecula (146.3)	2	2

*Best possible match for 2 cases was stage IVA instead of III.

**Table 2:** HPV and p16^INK4A^ results of all cases.

Case-case	p16^INK4A^ overexpression		HPV		Final HPV outcome
		PCR	PCR		
		(Amplicor)	(Linear Array)	FISH	
1A*	+	Present	HPV-33/52,33,35,58	Absent^§^	Positive
1B^†^	±	Present	Absent	Absent	Positive
2A	+	Present	Absent	Present	Positive
2B	+	Present	HPV-16	Present	Positive
3A	+	Present	HPV-16	Present	Positive
3B	−	Absent	Absent	Absent	Negative
4A	+	Present	HPV-16	Present	Positive
4B	−	Absent	Absent	Present^||^	Negative
5A	+	Present	Absent	Present	Positive
5B	−	Absent	Absent	Absent	Negative
6A	+	Present	HPV-16	Present	Positive
6B	−	Absent	Absent	Absent	Negative
7A	+	Present	HPV-16	Present	Positive
7B	−	Absent	Absent	Absent	Negative
8A	+	Present	HPV-16	Present	Positive
8B	−	Absent	Absent	Absent	Negative
9A	+	NO^‡^	NO^‡^	Present	Positive
9B	−	Absent	Absent	Absent	Negative
10A	+	Present	NO^‡^	NO^‡^	Positive
10B	−	Absent	Absent	Absent	Negative
11A	+	Present	NO^‡^	Present	Positive
11B	−	Absent	Absent	Absent	Negative
12A	+	NO^‡^	NO^‡^	Present	Positive
12B	−	NO^‡^	NO^‡^	Absent	Negative
13A	−	Absent	Absent	Absent	Negative
13B	−	Absent	Absent	Absent	Negative
14A	−	Absent	Absent	Absent	Negative
14B	−	Absent	Absent	Absent	Negative
15A	−	Absent	Absent	Absent	Negative
15B	−	Absent	Absent	Absent	Negative
16A	−	NO^‡^	NO^‡^	Absent	Negative
16B	+	NO^‡^	Absent	Absent	Negative

*A: nonsmoking and nondrinking.

^†^B: smoking and drinking.

^‡^Not obtained (for PCR tests, for example, due to a negative *β*-globin PCR).

^§^HPV-16-specific FISH probe.

^||^poor signal.

**Table 3:** Characteristics of all cases according to HPV status.

	HPV *n* (%)		
	Positive	Negative	
Variable	(*n* = 14)	(*n* = 18)	**P** value
Tobacco and Alcohol			*<0.001*
Nonsmoking and nondrinking	12 (86)	4 (22)	
Smoking and drinking	2 (14)	14 (78)	
p16^INK4A^ overexpression			*<0.001*
+	13 (93)	1 (6)	
−	0	17 (94)	
±	1 (7)	0	
Tumor location (ICD-code)			NS*
Base of tongue (141.0)	4 (29)	8 (45)	
Tonsil (146.0)	4 (29)	6 (33)	
Tonsillar fossa (146.1)	4 (29)	2 (11)	
Vallecula (146.3)	2 (15)	2 (11)	
Tumor			*NS*
T1	3 (21)	1 (6)	
T2	6 (43)	7 (39)	
T3	3 (21)	6 (33)	
T4	2 (15)	4 (22)	
			*NS*
T1-T2	9 (65)	8 (45)	
T3-T4	5 (35)	10 (55)	
Nodal involvement			*NS*
N0	4 (29)	7 (39)	
N1	3 (21)	4 (22)	
N2	7 (50)	7 (39)	
Stage			*NS*
II	2 (15)	4 (22)	
III	5 (35)	5 (28)	
IVA	7 (50)	9 (50)	
Year of initial diagnosis			*NS*
1982–1986	1 (7)	2 (11)	
1987–1991	2 (15)	3 (17)	
1992–1996	6 (43)	5 (28)	
1997–2001	2 (15)	5 (28)	
2002–2006	3 (21)	3 (17)	
Treatment modality			*NS*
Radiotherapy	6 (43)	6 (33)	
Chemotherapy + radiotherapy	2 (15)	0	
Surgery + radiotherapy	5 (35)	9 (50)	
Surgery	0	2 (11)	
Chemotherapy	0	1 (6)	
Supportive	1 (7)	0	
Tumor grade			*NS*
Moderate	7 (50)	4 (22)	
Poor	7 (50)	14 (78)	
Perineural growth			*NS*
Yes	4 (29)	2 (11)	
No	10 (71)	16 (89)	
Vasoinvasive growth			*NS*
Yes	3 (21)	1 (6)	
No	11 (79)	17 (94)	
Keratinization			*0.025*
Yes	3 (21)	11 (61)	
No	11 (79)	7 (39)	
Basaloid features			*0.039*
Yes	9 (65)	5 (28)	
No	5 (35)	13 (78)	
Tumor recurrence			*NS*
Yes	3 (21)	3 (17)	
No	11 (79)	15 (83)	
Second primary tumor			*NS*
Yes	1 (7)	4 (22)	
No	13 (93)	14 (78)	

*Nonsignificant.
